# Observation of persistent species temperature separation in inertial confinement fusion mixtures

**DOI:** 10.1038/s41467-020-14412-y

**Published:** 2020-01-28

**Authors:** Brian M. Haines, R. C. Shah, J. M. Smidt, B. J. Albright, T. Cardenas, M. R. Douglas, C. Forrest, V. Yu Glebov, M. A. Gunderson, C. E. Hamilton, K. C. Henderson, Y. Kim, M. N. Lee, T. J. Murphy, J. A. Oertel, R. E. Olson, B. M. Patterson, R. B. Randolph, D. W. Schmidt

**Affiliations:** 1 Los Alamos National Laboratory, P.O. Box 1663, MS T087, Los Alamos, NM 87545 USA; 2 Laboratory for Laser Energetics, University of Rochester, 250 E. River Rd., Rochester, NY 14623 USA; 3 Present Address: Laboratory for Laser Energetics, University of Rochester, 250 E. River Rd., Rochester, NY 14623 USA

**Keywords:** Nuclear fusion and fission, Laser-produced plasmas

## Abstract

The injection and mixing of contaminant mass into the fuel in inertial confinement fusion (ICF) implosions is a primary factor preventing ignition. ICF experiments have recently achieved an alpha-heating regime, in which fusion self-heating is the dominant source of yield, by reducing the susceptibility of implosions to instabilities that inject this mass. We report the results of unique separated reactants implosion experiments studying pre-mixed contaminant as well as detailed high-resolution three-dimensional simulations that are in good agreement with experiments. At conditions relevant to mixing regions in high-yield implosions, we observe persistent chunks of contaminant that do not achieve thermal equilibrium with the fuel throughout the burn phase. The assumption of thermal equilibrium is made in nearly all computational ICF modeling and methods used to infer levels of contaminant from experiments. We estimate that these methods may underestimate the amount of contaminant by a factor of two or more.

## Abstract (web summary)

The influence of contaminants is one of the factors hindering self-sustained thermonuclear burn in inertial confinement fusion. Here, the authors present evidence, through simulations and experiments, that contaminants do not fully reach thermal equilibrium, and thus their amount is usually underestimated.

## Introduction

Inertial confinement fusion (ICF) involves the compression of capsules containing deuterium and tritium (DT) with the goal of achieving self-sustaining thermonuclear burn^1^. The performance of capsule implosions is limited by the introduction of contaminant material that mixes with the fuel^2–6^ through interfacial instabilities^7^ and engineering features^8,9^. We use the term mixing broadly, to include the introduction of contaminant (non-hydrogenic) material into the fuel region of a capsule implosion. This includes the limiting cases of contaminant that is atomically mixed with the fuel as well as contaminant “chunks” that penetrate the fuel region as macroscopic fingers, jets, or “meteors”. This contaminant degrades yield by enhancing conductive and radiative losses, as well as displacing fuel. ICF experiments have recently achieved an alpha-heating regime^10,11^, in which fusion self-heating is the dominant source of yield, by reducing the susceptibility of implosions to the instabilities that inject contaminant mass.

It has been argued^5,6^ that material mixed into hot, compressed fuel equilibrates rapidly with the hot spot due to rapid inter-particle collisions, so that the equilibration time can be neglected. This assumption is essential to estimates of mix mass from X-ray emission^3–6^ that are frequently used in the literature as well as our understanding of how contaminant degrades yield. It also implies that chunks of contaminant rapidly atomize, which has not been demonstrated. In the literature, the impact of mixing has focused on radiative losses associated with hot contaminant. Nevertheless, by effectively increasing the surface area of the hot spot and introducing large temperature gradients, cold contaminant enhances conductive losses, which can be just as significant. Whether conductive or radiative losses are dominant depends on the local hot spot conditions, quantified by the temperature and areal density (see, e.g., ref. ^12^) and the conduction losses will be amplified by the existence of cold chunks of contaminant. It is therefore crucially important to understand the amount of both cold and hot contaminant in fuel regions.

Species temperature separation arises for several reasons. Hot spot ignition designs for the National Ignition Facility (NIF) require carefully timed shocks to heat the fuel layer, and hence the ablator, less than the low-density vapor. In general, temperature separation arises from different specific heats of initially separated materials, causing hydrogenic fuel to heat more than contaminant. Thermal equilibration between separated species can occur via electron thermal conduction (ion conduction is a factor $\sqrt{{m}_{\text{e}}/{m}_{\text{i}}}$ smaller) or local collisions after atomically mixing—i.e., diffusion or convection—and the latter process is much faster. The timescale at which a hot *T*_e_ = 1 keV mixing region with a 50*μ*m extent equilibrates by electron thermal conduction can be estimated, assuming full ionization and using Spitzer’s conductivity^13^, as *τ* ≈ *l*^2^*ρC*_V,e_*κ*^−1^ ≈ 1.6 ns. Therefore, thermal equilibration between initially separate species is dependent on the development of atomic mixing, which varies depending on implosion details. Simulations resolving the mixing processes in warm OMEGA implosions indicate that atomic mixing continues to develop through the burn phase^14^. For more general hydrodynamically unstable flows, one can apply theory from ref. ^15^ to estimate the time t^*^ for the flow to achieve a mixing transition^16^, in which the flow drives a rapid development of atomic mixing,

1 $${t}^{* }\ge \delta {u}^{-1}10{0}^{2}{C}_{\,\text{D}}^{-2}{\text{Re}}^{* -1/2},$$

where Re^*^ = 1.6 × 10^5^, *δ* is the experimental enclosure lengthscale, *u* is the characteristic velocity, and *C*_D_ is a diffusion layer coefficient (here, ${C}_{\text{D}}=\sqrt{15}$). For high-yield NIF capsule implosions^11^, t^*^ ≈ 6−12 ns, comparable to implosion timescales. Since many instabilities arise late in the implosion, chunks of contaminant have the potential to remain unmixed and hence unequilibrated with the fuel during the burn phase.

We report the results of experiments and highly detailed and resolved 3D simulations that provide evidence that for a wide range of initial conditions, mixing regions in inertially confined plasmas—i.e., regions where contaminants have mixed with hydrogenic fuel—maintain chunks of contaminants that do not achieve thermal equilibrium (for both ions and electrons) with the fuel during ICF implosions. We will demonstrate that as a consequence, models used to estimate contaminant mass through enhanced X-ray bremsstrahlung emission^2–6^ may underestimate the amount of contaminant by a factor of two or more. Given the physical processes involved, a corollary of our observation is that atomic mixing between fuel and contaminant develops faster than equilibration through conduction and that both processes can be slower than the implosion. This observation is critically important to ICF because of the strong sensitivity of contaminant bremsstrahlung radiative emission, the dominant energy loss mechanism, and DT thermonuclear reactivity ${\overline{\sigma v}}_{\text{DT}}$ to temperature variations. The power of bremsstrahlung emission ${P}_{\text{br}}\propto \sqrt{{T}_{\text{e}}}$^12^, where *T*_e_ is the electron temperature, and ${\overline{\sigma v}}_{\text{DT}}\propto {T}_{\text{i}\,}^{\alpha }$, where *T*_i_ is the ion temperature, with 3 ≲ *α* ≲ 7 for ICF-relevant temperatures. The introduction of cold contaminant into the hot spot also enhances conductive losses, due to the presence of temperature gradients inside the hot spot as well as enhancing the surface area of the hot spot boundary. Temperature separation has previously been observed in experiments designed to maximize kinetic effects^17^ as well as between species in fuel regions in more hydrodynamic implosions^18^. The primary advancement of our work is that we consider conditions in compressed mixing regions containing both carbon and fuel, conditions that have been observed to minimize kinetic effects^19–21^. Our results suggest that there is margin to improve the prospects for ignition by improving target quality to reduce the magnitude of imperfections that seed these instabilities or by improving tolerance to these asymmetries.

## Results

### Description of experiments and context

**Fig. 1:**
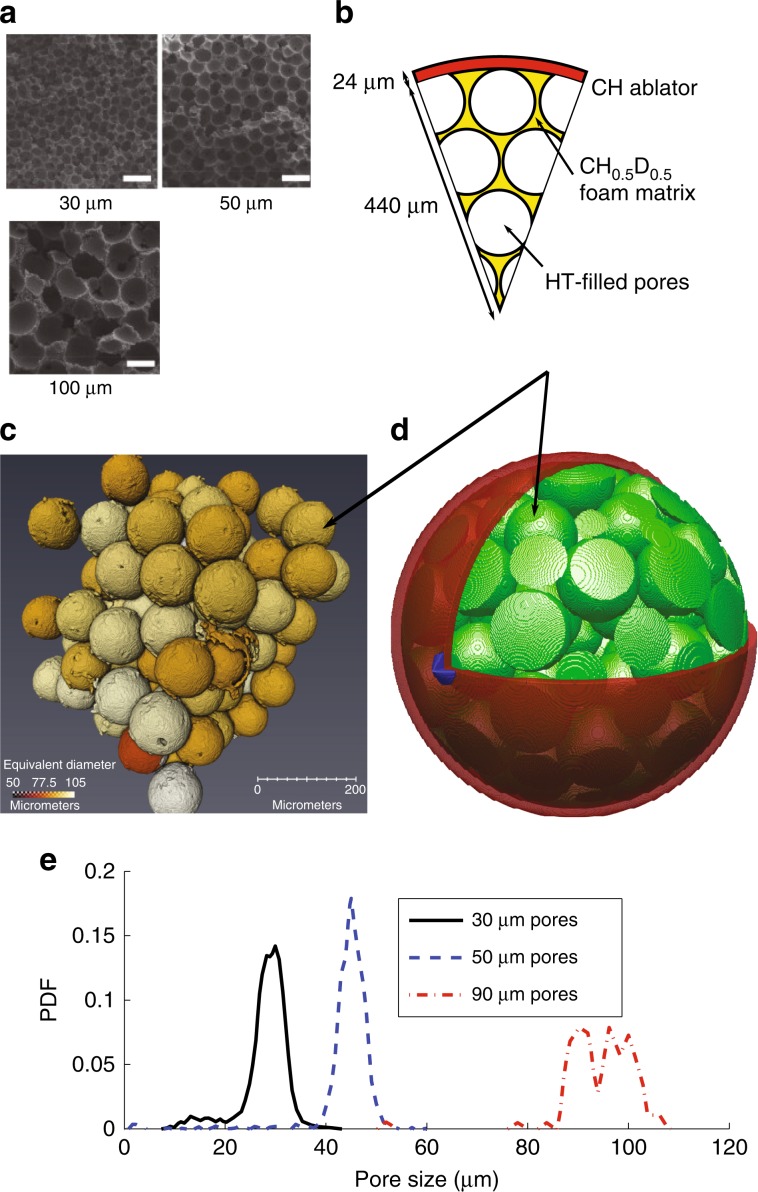
Initial configuration of experiments and simulations. **a** Scanning electron microscope image of engineered foams^44^ showing different macro-pore sizes. **b** Capsule diagram; foam matrix is open cell, containing sub-micron pores that are also filled with HT. **c** Visualization of pores (solid spheres are macro-pores), colored by diameter, for 90 μm pore foam sample. **d** Visualization of initial conditions for 3D simulation with 90 *μ*m pores; red: shell, green: foam pores (filled with HT), blue: glue spot for target mount. **e** Probability distribution function (PDF) of pore diameters.

We report the results of novel separated reactant ICF experiments designed to help to understand the behavior of contaminant mass during ICF implosions, along with detailed and highly-resolved three-dimensional (3D) simulations that are in good agreement with the experimental data. Our experiments explore the limiting case where chunks of contaminant are present in the initial conditions, allowing the maximum possible time to atomically mix and equilibrate with the fuel. Our experiments involve the compression of capsules (Fig. 1b) containing deuterated open-cell foams whose pores are filled with hydrogen and tritium, and we control the initial conditions by varying the foam pore sizes. Contaminant and fuel are initially physically separated and heat to different temperatures due to this separation as well as their different specific heats. As the implosion progresses, hydrodynamic processes lead to atomic mixing of these materials, resulting in a complex distribution of materials containing regions of pure chunks of contaminant or fuel and atomically mixed regions. As a result of the process of atomic mixing, fuel and contaminant in atomically mixed regions locally achieve thermal equilibrium. Our experiments uniquely address the persistence of chunks and whether these chunks remain in thermal equilibrium with the fuel. Furthermore, our results indicate that the observed phenomena can be explained by appealing to well-known physical processes—hydrodynamic instabilities, which lead to mixing and local equilibration, combined with thermal conduction, which brings materials into thermal equilibrium over larger scales—and the time-scales over which they operate. These timescales have previously only been addressed theoretically.

We will demonstrate reactant temperature separation in experiments by contradiction. Assuming the reactants are in thermal equilibrium, *T*_DT_ = *T*_DD_, and the DT∕DD yield ratio is determined by the extent to which the reactants are atomically mixed. However, our experimental data lie above this theoretical maximum, so this assumption cannot be correct. To further support this conclusion, reactant temperature separation is directly observed in simulations that are in agreement with the experimental observables. We have evaluated several alternative explanations, including kinetic effects, anomalously high ion temperatures, target mischaracterization, and complete atomic mixing, and these cannot plausibly explain the data.

The observation of species temperature separation in ICF mixing regions has important consequences for our understanding of implosions. Models used to estimate contaminant mass through enhanced X-ray bremsstrahlung emission assume thermal equilibrium between contaminant and DT^2–6^. Such models underpredict the amount of contaminant by overestimating the emission of the contaminant. Due to the difficulty of probing the relevant timescales and lengthscales, our understanding of ICF implosions relies on simulations, which nearly universally assume mixtures to be in thermal equilibrium through modeling choices. Only with significant time on today’s largest supercomputers is it possible to accurately simulate the inherently 3D hydrodynamics^22^ and coupled physics at the sub-micron scales where mixing occurs. Therefore, this has only been done a few times^7,14,19,23,24^. Instead, mixing is either ignored, enforced in initial conditions, or modeled with a dynamic mix model, e.g., Reynolds-Averaged Navier-Stokes (RANS). As presently implemented, these force mixtures into thermal equilibrium (i.e., they assume negligible equilibration timescales regardless of how chunky the mixture is). RANS models can be coupled to reaction rate equations^25^ as for reactive flows^26,27^, e.g., flames, detonations, and combustion, where equilibration timescales are much shorter than reaction timescales. This works in the absence of reactivity fluctuations^28^. However, our results indicate that fluctuations are present and persistent in ICF mixing layers, so that these methodologies are incomplete in the context of ICF. In ICF simulations, temperature gradients cause deviations between DT and DD reaction burn-weighted ion temperatures (BWTI), yet larger deviations are observed experimentally^29^. Thermal fluctuations caused by complicated material distributions with unequilibrated contaminants could contribute to this discrepancy. This could also explain why RANS simulations consistently require unphysical adjustments to reproduce the results of separated reactants experiments^30,31^. Additionally, thermal fluctuations could seed velocity gradients that broaden the neutron spectrum, resulting in artificially high measured ion temperatures^32^.

Another open question in ICF has been whether hydrodynamic approximation of the true system is sufficient to capture the evolution of contaminant in an ICF hot-spot. Our experiments, which involve complex and evolving material distributions, provide a test bed, and our high-resolution 3D simulations, which are necessary to test this, capture the experimental observables, providing evidence that the hydrodynamic approximation remains valid.

To test the impact of our observation, we performed 2D simulations^9,33,34^ of a high-yield implosion^11^ including the fill tube, a source of chunks of contaminant. In simulation, 160 ng of contaminant mass is injected into the hot spot, and its temperature remains 2.5 keV lower than that of the DT. However, applying experimental methods for calculating contaminant mass using continuum emission from ref. ^5,6^ to simulated X-ray self-emission produces an estimate of 80 ng. The discrepancy arises because the model assumes the contaminant is radiating at the temperature of the fuel, which is equivalent to approximately doubling the contaminant emissivity. This effect will also have implications for interpretation of the line emission from contaminant mass. For example, temperature fluctuations such at this will likely be associated with density fluctuations, which have been demonstrated to significantly affect emissivity^35^.

Our experiments, performed as part of Los Alamos National Laboratory’s (LANL) MARBLE campaign^36^, are separated reactants experiments in which deuterium resides in contaminant material instead of fuel, so DT reactions occur only as a consequence of atomic mixing between the contaminant and the fuel. Previous separated reactants experiments^14,30,31,37–41^ included deuterium in the shell, whereas our capsules contain open-cell deuterated polystyrene (CH_0.5_D_0.5_) foams^42–44^ whose pores are filled with hydrogen and tritium (HT). This represents the limiting case in which chunks of pre-mixed material exist in the hot spot before it forms and are given the maximum possible time to atomically mix and equilibrate with the fuel. Foams are engineered with fixed macro-pores sizes^44^ of 30, 50, and 90 μm, in addition to the sub-micron pores that exist throughout the open-cell foam matrix. The macro-pores seed hydrodynamic instabilities, primarily the Richtmyer-Meshkov instability^45–48^, which induce mixing between the foam and HT. The RMI is known to exhibit sensitivity to and memory of initial conditions both in fluid^49^ and plasma^50^ regimes. Larger pores are expected to produce mixing that is more “chunky” (and hence produce a lower DT∕DD yield ratio) whereas smaller pores produce more atomic mix (hence a higher DT∕DD ratio). Ten capsule implosions were directly driven at the Laboratory for Laser Energetics’ (LLE) OMEGA laser facility with a 1ns square pulse using 26kJ of laser energy; another 12 implosions were performed on a separate shot day that produced consistent results. In simulations, conditions in the burn region are similar to those observed in mixing regions in simulations of current high-yield implosions^11^.

### Simulation details

Simulations used LANL’s xRAGE^9,51^, applying methodology for simulating direct drive implosions detailed in ref. ^14^. xRAGE features adaptive mesh refinement (AMR), making it ideal for resolving the foam pore structure. xRAGE has previously been used to successfully model experiments involving foams^33,34,36,52–57^. Simulations included shell thickness variations and the target mount. Example initial conditions are visualized in Fig. 1d. The use of 3D was necessitated by foam geometry and hydrodynamically unstable flows^22^. To resolve mixing processes, simulations used a maximum resolution of 0.25 μm. This resolves the Kolmogorov lengthscale^58^ *λ*_K_ ≈ 0.5 μm for these experiments (using viscosity formula in ref. ^59^). Previous 3D simulations of separated reactants experiments^14^ and reduced-dimension grid resolution studies suggest that this resolution is sufficient. Due to the high carbon content of the implosion, it was not necessary to explicitly model diffusion and viscosity^19–21^. Instead, an implicit large eddy simulation (ILES) strategy^60^ was employed. Using this methodology, diffusion and viscosity are implicitly modeled by numerical filtering; this approach has been successfully used to model separated reactants ICF implosions^14^. Three simulations, using a total of 510 million CPU hours, were performed on Lawrence Livermore National Laboratory’s Sequoia supercomputer. The total cell count ranged from 0.3 to 3.0 billion. Axisymmetric 2D simulations showed substantial differences from 3D simulations, arising from differing surface area to volume ratios of the pores, leading to differences in pore collapse and shock dynamics, indicating the inadequacy of 2D.

**Fig. 2:**
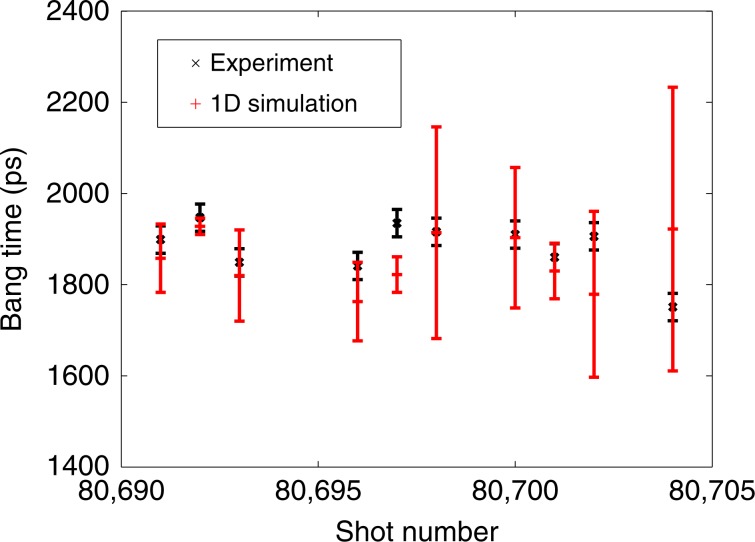
Comparison of simulated and experimental bang times. Bang times (time of peak neutron production) for all ten capsule implosions compared to 1D simulation results. 1D simulations account for variations in HT fill pressure, as-shot laser power history, and capsule ablator thickness. The simulation error bars indicate the effect of measured ablator thickness variances, which ranged from 0.5 to 8.7 μm; the average ablator thickness varied from 22.0 to 26.2 μm. The simulated error bars were calculated based on the standard deviation of 1D simulations performed at the extremes of measured ablator thickness and ablator thickness variances. The experimental bang times were determined from a neutron temporal diagnostic and the error is based on the response time of this instrument^61^.

We performed 1D simulations of all ten experiments including as-shot drives, HT fill pressures, and capsule geometries in order to evaluate our ability to model the shock and implosion trajectories. Due to the complex target design, variations in these parameters were large. Fill pressures varied from 6.2 to 8.6 atm, ablator thickness varied from 22.0 to 26.2 μm, and ablator thickness variances ranged from 0.5 to 8.7 μm. These parameters affect the shock speed through the foam as well as the implosion timescale. Therefore, the ability to capture the effects of varying these parameters is a strong constraint on simulations. By varying only these parameters according to measured values, our 1D simulations captured the measured bang time (time of peak neutron production) variations within experimental error for all but one of the ten shots. This is shown in Fig. 2, where error bars on the simulations indicate variation due to capsule ablator thickness variance.

Several companion experiments were performed to evaluate simulations, including capsule implosions with deuterated methane, deuterated foams with and without hydrogen gas, and CH foams and deuterium gas. Cylindrical experiments were also performed to enable X-ray radiography of shock transit through various foams and pore collapse. For capsule implosions, simulations accurately captured experimental yield and bang time trends. For cylindrical experiments, simulations accurately captured shock and pore dynamics.

### Comparison between simulations and experiments

**Fig. 3:**
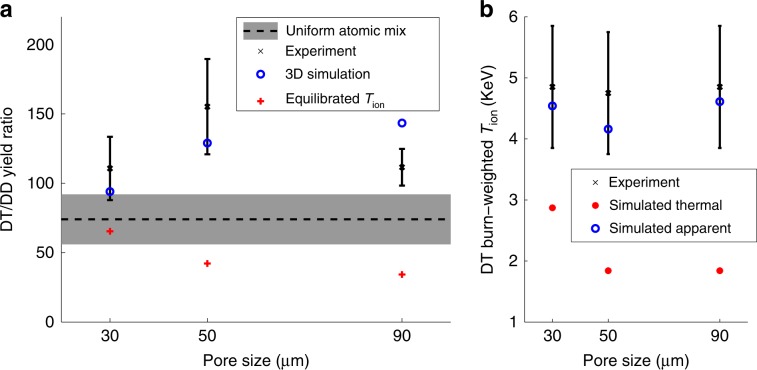
Comparison between 3D simulations and experiments. **a** DT∕DD reaction yield ratios corrected for as-shot variations. The black ‘x' symbols denote the experimental values with the solid black lines indicating the experimental measurement error, determined based on the method described in ref. ^62^. The black dashed line indicates the maximum theoretical yield ratio (Eq. (3)) for uniform atomic mix and the shaded region indicates uncertainty. This uncertainty is the maximum and minimum possible values calculated based on measurement uncertainties of foam densities, foam composition, fill pressure, and fill gas composition. Measurement errors are quantified in the Methods section and described in^42–44,63–65^. The red plus symbols indicate values that would have resulted from simulations if the reactants were in thermal equilibrium. **b** Comparison of experimental burn-weighted *T*_i_, simulated thermal burn-weighted *T*_i_ from 3D simulations, and simulated apparent (including fluid motion corrections based on ref. ^66^) burn-weighted *T*_i_ from 3D simulations. A forward-fit approach using a relativistic energy distribution is used to analyzed the neutron time-of-flight signal to infer the neutron-average ion temperature, and the error bars are determined from the statistical uncertainty in this fit to the data^67^.

**Fig. 4:**
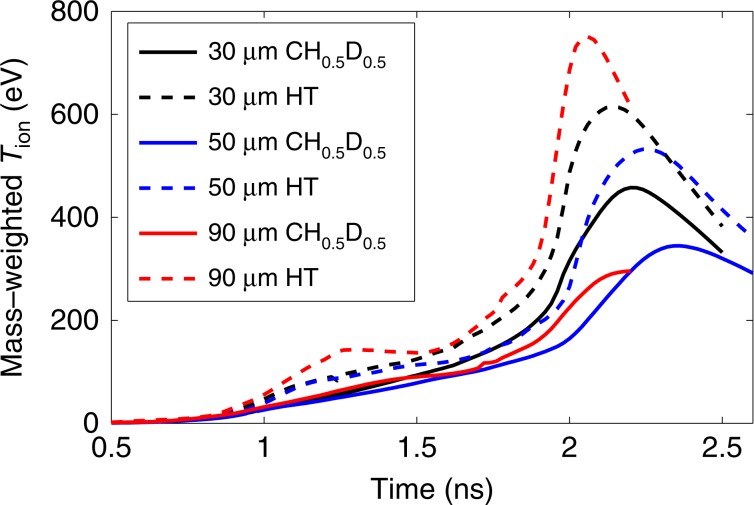
Comparison of simulated species temperatures. Mass-weighted ion temperatures for each reacting material in 3D simulations with varied pore size Data is included for simulations with 30 μm (black), 50 μm (blue), and 90 μm (red) pore sizes. The temperatures for the CH_0.5_D_0.5_ foam matrix use solid lines and the temperatures for the HT gas fill use dashed lines. The HT temperatures are consistently higher than the CH_0.5_D_0.5_ temperatures throughout the compression- and burn-phases of the implosion.

The primary observable in experiments is the DT∕DD neutron yield ratio, which measures the amount of atomic mixing. Experimental results, including yield ratios and DT burn-weighted *T*_i_ (BWTI) from 10 shots, are presented along with results from 3D simulations in Fig. 3. Yield ratios are normalized by

2 $$\frac{\,{\rm{Average}}\, {\rm{1D}}\, {\rm{simulated}}\, {\rm{yield}}\, {\rm{ratio}}}{{\rm{Shot}}\, {\rm{1D}}\, {\rm{simulated}}\, {\rm{yield}}\, {\rm{ratio}}}$$

to account for variation of the laser pulse, fill pressure, and ablator thickness. The experimental results exhibit no significant dependence on pore size. Apparent temperatures from 3D simulations, including fluid motion corrections^66^, are plotted in Fig. 3b and are in good agreement with experiment. If we assume the reactants are in thermal equilibrium, observed yield ratios are consistently higher than the theoretical maximum. We calculate the maximum yield ratio (the uniform atomic mixed limit) assuming thermal equilibrium by dividing the DT reaction rate formula by the DD reaction rate formula and noting that atomic mix maximizes the former and minimizes the latter, hence maximizing the ratio. The resulting theoretical maximum is given by

3 $$\frac{2\ {n}_{\text{T}}\ {\overline{\sigma v}}_{\text{DT}}({T}_{\text{i}})}{{n}_{\text{D}}\ {\overline{\sigma v}}_{\text{DD}}({T}_{\text{i}})}=74\pm 18,$$

where *n*_D_ and *n*_T_ are the number of deuterons and tritons, respectively. This estimate is also consistent with 1D simulations where atomic mixing is enforced in the initial conditions. Our assumption that the reactants are in thermal equilibrium implies that the BWTI is the same for the DT and DD reactions. Therefore, we have evaluated this at the experimental DT BWTI, and this dominates the uncertainty. Nevertheless, it should be noted that our results do not depend on which temperature is used for this estimate: even making the extreme assumption that *T*_i_ = 11.6 KeV to maximize the reactivity ratio, the resulting estimate is 105. Experiments and simulations are consistent with DT reactions occurring at a higher temperature than DD reactions. In simulations, the reactant temperature separation can be directly observed (Fig. 4). This results in DT BWTIs that exceed the DD BWTIs by  ≈0.25 keV, which is sufficient to raise the yield ratio by 30–40%. This is larger than the reaction BWTI separation that arises due to thermal gradients in a fully atomically mixed hot spot. For comparison, we include results that would have been produced by the 3D simulations if the reactants were in thermal equilibrium (labeled “Equilibrated *T*_ion_”). We calculated this by integrating the reaction rate equation spatially and temporally over the burn region using the instantaneous burn-weighted *T*_i_ to calculate the DT and DD reactivities rather than the local ion temperatures. The resulting yield ratios are below the theoretical maximum and decrease with increased pore size, dominated by reactant spatial distribution.

**Fig. 5:**
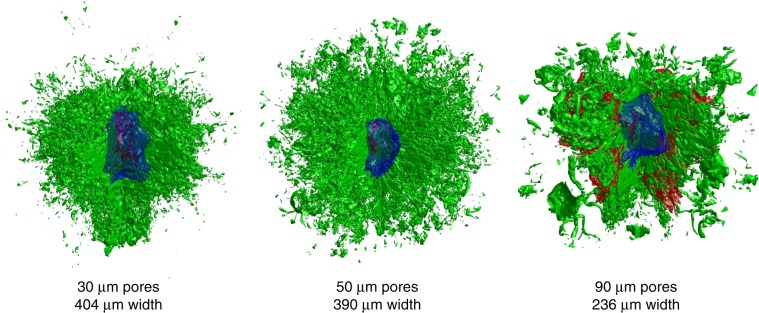
Simulation visualizations. Visualizations of the shock (red), pore interfaces (green), and hot spot boundary (blue) at bang time for the three different initial pore sizes. A quadrant has been cut out of the shock and pore data in order to aid in the observation of internal features.

Simulated mass-weighted temperatures are in Fig. 4 (results for *T*_e_ are similar). In all cases, the gas heats more than the foam matrix. The foam temperature peaks later than the HT temperature due to convective heating: vortical flows atomically mix hot HT with colder foam material, heating the latter. The level of separation between temperatures is proportional to pore sizes: simulations with smaller pores exhibit more atomic mixing, enabling local temperature equilibration. The 30 μm pore simulation exhibits the most atomic mix, as quantified by the spatial distribution of HT mass, whereas the 50 and 90 μm pore simulations demonstrate decreasing levels of atomic mix. This behavior is visualized in Fig. 5, where we visualize material distributions at bang time, where it is possible to observe the persistence of larger scale regions of pure HT in implosions with larger pores.

MARBLE experiments underway at the NIF have demonstrated similar trends to the data presented above. With more laser power available to drive larger capsules, it is possible to obtain more implosion data, including X-ray imaging and BWTIs for both reactions. Results exhibit an increase in BWTI with increased pore size consistent with the results in this paper. Results also indicate a small increase in DT∕DD yield ratio with increased pore size, consistent with temperature separation between reactants.

## Discussion

We evaluated alternative hypotheses for why experimental yield ratios lie above the theoretical maximum, including kinetic effects, anomalous temperatures highly discrepant from the computed values, mischaracterization of targets and/or fill resulting in anomalously high tritium concentrations relative to deuterium, and the possibility that the reactants are atomically mixed before bang time. None of these things can plausibly explain the experimental data. Unmodeled kinetic effects are unlikely to have a large enough effect to explain the results because of the high ion densities and carbon presence. As a result, the Knudsen number inside the burn region, which quantifies the validity of continuum approximations, can be estimated as $\,\text{Kn}\,=\frac{\lambda }{L}\approx 0.001$, where *λ* is the ion mean free path and *L* is a physical length scale (here, the temperature gradient scale length is used). Nevertheless, kinetic simulations of small regions under conditions relevant to MARBLE implosions show increases to DT fusion rates from non-Maxwellian distributions and relative drifts between species, and decreases from Knudsen-layer losses. These have a complex integrated effect on the results that is currently being explored^68^. Furthermore, these results are not inconsistent with our conclusions. Discrepant temperatures cannot explain the results since, by setting *T*_i_ = 11.6 KeV into the theoretical maximum given by Eq. (3) to maximize ${\overline{\sigma v}}_{\text{DT}}({T}_{\text{i}})/{\overline{\sigma v}}_{\text{DD}}({T}_{\text{i}})$, the resulting yield ratio, 105, is still too low to explain the experimental data. Target mischaracterization would require density or pressure deviations greater than five times the measurement error (quoted in the Methods section) to achieve the experimental results. Finally, if the reactants were completely atomically mixed at bang time, the experimental yields would have to fall within the estimate given in Eq. (3) (which is also consistent with simulations where atomic mix is enforced). However, the data lie well above this estimate.

In summary, we have performed novel separated reactants experiments along with detailed high-resolution 3D simulations, which indicate that species temperature separation is present and persistent at conditions relevant to mixing regions in high-yield ICF experiments. In experiments, we infer this from the ratio of DT∕DD neutron yields, which is higher than can be explained when reactants are in thermal equilibrium. Experimental results, including yield ratios, are in agreement with simulations in which species temperature separation is directly observed. Furthermore, simulations and plasma theory indicate that separations persist throughout the burn phase. These results indicate that the amount of contaminant mass in the hot spots of high-yield implosions is routinely underestimated, since methods for estimating this mass assume thermal equilibrium between contaminant and fuel. This suggests that there is significant margin for improving the performance of capsule implosions through decreasing the magnitude of capsule asymmetries or adjusting designs to make them more robust to hydrodynamic instabilities.

## Methods

### Targets

Capsules consisted of deuterated open-cell polystyrene (CH_0.5_D_0.5_) foams with 430 *μ*m radius surrounded by polystyrene (CH) shells with 24 μm thickness (see Fig. 1). For the present experiments, foams were produced with fixed macro-pore sizes of 30, 50, and 90 μm. The process for producing the engineered pore foams was developed in ref. ^44^. The engineered pore foams are constructed by placing hollow spherical SiO_2_ particles of the desired pore size in an aerogel precursor. Once the matrix has polymerized, the SiO_2_ particles are chemically leached out. The matrix foam is prepared such that the final bulk density is approximately 30 mg cm^−3^. The matrix itself also includes sub-micron pores. The engineered pores were characterized using X-ray microscale tomography^63,64^ to determine the distribution of pore sizes and void fractions. The void fractions for all foams was measured to be 49 ± 2%. The atomic compositions of the foam were measured using carbon 13 nuclear magnetic resonance spectroscopy, indicating that the foams are 50 ±  1% carbon, 25.5 ± 1% hydrogen, and 24.5 ± 1% deuterium. Density measurements were made gravometrically using machined samples of the foams with known volumes, and variations among different samples were evaluated from transmission measurements using a narrow-band X-ray source^65^. The bulk density for all foams was measured to be 33.0 ± 0.5 mg cm^−3^. The foams are machined into 860 μm-diameter spheres^43^ and placed inside machined Rexolite step-jointed hemi-shell halves^42^, which were then glued and machined into 900 μm-diameter spheres, which were then coated with 3 μm of glow discharge polymer (GDP). This procedure produced capsules with average shell thicknesses ranging from 22.0 to 26.2 μm and shell thickness variances ranging from 0.5 to 8.7 μm.

### Experiments

Experiments were performed at the University of Rochester’s OMEGA laser facility. Capsules were imploded in a direct-drive configuration using all 60 beams of the OMEGA laser, delivering 25 kJ of energy in a 1ns square pulse. The laser energy delivery was within 3% of the requested value for all shots. Based on the availability of capsules, the implosions included three capsules with 30 μm pores, five capsules with 50 μm pores, and two capsules with 90 μm pores. The capsules were diffusion filled with a requested 10 atm of HT gas, which occupies both the foam macro-pores and the sub-micron pores in the matrix. Due to leakage, the fill pressure at shot time varied from 6.2 to 8.6 atm.

**Fig. 6:**
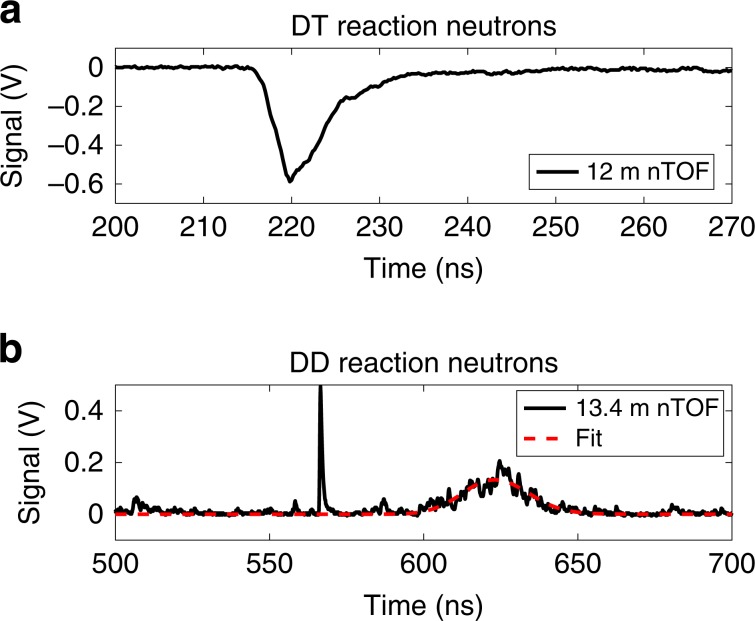
Neutron time-of-flight data frome experiment. The neutron time-of-flight signal (red) from shot 80691 is obtained from two separate neutron time-of-flight (nTOF) diagnostics positioned 12 and 13.4-m, respectively, from target chamber center (TCC). A forward-fit approach (dashed-red), shown for the DD reaction, using a relativistic energy distribution is used to analyzed the time-of-flight signal to infer the yield and neutron-average ion temperature.

Because of the large amount of carbon in the hot spot, which gives the fuel a large heat capacity and allows for significant radiative losses, the experimental yields were very low compared to typical gas-filled capsules imploded on OMEGA, producing yields of only 1.3  × 10^9^ to 2.3 × 10^9^ DT neutrons and 7.8 × 10^6^ to 2.2 × 10^7^ DD neutrons. Because of this, the neutron diagnostic data were too noisy to extract typically reported quantities such as the time-dependent neutron production rate. Bang times (the time of peak neutron production) and BWTI were manually extracted from the data instead of using typical automated processes. An example of the analysis of a neutron time of flight (nTOF) signal is shown in Fig. 6.

### Simulations

Simulations were performed using Los Alamos National Laboratory’s (LANL) xRAGE radiation-hydrodynamics code^9,51^. xRAGE solves the Euler and coupled equations in an Eulerian reference frame utilizing square/cubic cells and features AMR, which makes it ideal for performing simulations with a resolved pore structure. The hydrodynamics solver is a custom approximate Godunov-type solver for the Euler equations similar to that of Harten-Lax-van Leer^69^. The radiation transport equations are solved using a multi-frequency gray diffusion approximation^70^. Simulations employ LANL OPLIB opacity data^71^ through the TOPS code^72^ and LANL SESAME tabular equation of state data^73^. Electron and ion thermal conductivities are based on the formulae of Lee and More^74^ with modifications^75^. Laser ray-tracing is performed using the Mazinisin laser package^76^ developed by the University of Rochester’s Laboratory for Laser Energetics, which has been integrated into the xRAGE code.

The simulation strategy used to model these direct-drive experiments is substantially similar to that outlined in^14^. One- and two-dimensional simulations were driven by the laser ray-tracing algorithm. In 3D simulations, the capsule is driven by an energy source designed to mimic the laser energy deposition as accurately as possible. The energy source is determined by dividing the shell into 1 μm increments and equating the energy deposition to that performed by a reduced-dimension simulation using the laser ray-trace. This procedure results in nearly identical results to simulations with the laser ray-trace in 1D, as measured by the material and radiation energies, shell trajectory, shock front position, shell thickness, and integrated burn metrics^14^. Simulations are performed with a maximum AMR resolution of 0.25 μm. Using the plasma viscosity formula in ref. ^59^, the Kolmogorov lengthscale *λ*_K_^58^, the lengthscale at which viscosity becomes dominant, can be estimated as *λ*_K_ ≈ 0.5 μm for the flows in these experiments. Therefore, in order to adequately resolve mixing processes, simulations were performed using AMR with a maximum resolution of 0.25 μm, which was adequate to resolve mixing processes in previous 3D simulations of separated reactants experiments^14^. Grid resolution studies were also performed in 1D and 2D; the results suggest that this resolution is sufficient to achieve grid convergence for these simulations.

Diffusion and viscosity are not explicitly modeled in simulations. We believe this is justified due to the relatively high carbon content of the hot spot, which limits the hot spot temperature and increases the average ion charge *Z*, both of which tend to increase the value of the plasma coupling parameter *Γ* and hence limit both the viscosity and the diffusion^19–21^. Furthermore, simulations of previous separated reactants experiments with explicitly modeled plasma diffusion and viscosity^14^ did not produce experimentally distinguishable differences in mixing quantities. In 3D, the simulations without explicitly modeled diffusion and viscosity can be considered ILES^60^, in which large energy containing structures are resolved and smaller structures are filtered out by the numerics.

Three 3D simulations were performed on Lawrence Livermore National Laboratory’s Sequoia supercomputer. One simulation was performed for each pore size used in experiment. The total cell count in the simulations ranged from 0.3 to 3.0 billion cells (the cell count varies during the computation due to the use of AMR) and each simulation ran for approximately 193 days using 49,152 processors, using a total of 510 million CPU hours between the three simulations.

The 3D simulations included accurate models for the foam geometry as characterized. This was done by sampling the distribution functions calculated from foam sample (Fig. 1e). Simulations also included measured shell thickness variations and the glue spot from the target mount. An example of resulting initial conditions for the 90 μm pore 3D simulation is visualized in Fig. 1d. It should be noted that the joint between the capsule hemispheres was not modeled and the relative orientation of the glue spot, joint, and exact pore positions is unknown for this set of shots.

In order to evaluate our ability to accurately model foam-filled capsule implosions, several companion experiments were performed on the OMEGA laser facility. These included the implosions of capsules filled with deuterated methane, capsules containing deuterated foams with and without hydrogen gas, and capsule implosions including CH foams and deuterium gas. In addition, cylindrical experiments were performed to enable X-ray radiography of shock transit through various foams as well as pore collapse. In all the capsule implosions, xRAGE simulations accurately captured experimental trends in terms of yields and bang times. In the case of the cylindrical experiments, simulations accurately predicted shock propagation and pore dynamics.

## Data Availability

The data that support the findings of this study are available from the corresponding author upon reasonable request.
